# Supplementary material for: A fault-intrusion-tolerant system and deadline-aware algorithm for scheduling scientific workflow in the cloud
Source: PeerJ Comput Sci. 2021 Nov 2;7:e747. doi: 10.7717/peerj-cs.747 (PMC8576554; doi:10.7717/peerj-cs.747)
Supplement: Supplemental Information 6 [file peerj-cs-07-747-s006.zip › Myproject1.1/docs/org/cloudbus/cloudsim/network/datacenter/class-use/NetworkDatacenter.html]

Uses of Class org.cloudbus.cloudsim.network.datacenter.NetworkDatacenter


JavaScript is disabled on your browser.


- Overview
- Package
- Class
- Use
- Tree
- Deprecated
- Index
- Help

- Prev
- Next

- Frames
- No Frames

- All Classes

## Uses of Class org.cloudbus.cloudsim.network.datacenter.NetworkDatacenter

- Packages that use NetworkDatacenter

  | Package | Description |
  | --- | --- |
  |  |  |
  | --- | --- |
  | org.cloudbus.cloudsim.network.datacenter |  |
- - ### Uses of NetworkDatacenter in org.cloudbus.cloudsim.network.datacenter

    Fields in org.cloudbus.cloudsim.network.datacenter declared as NetworkDatacenter

    | Modifier and Type | Field and Description |
    | --- | --- |
    |  |  |
    | --- | --- |
    | `NetworkDatacenter` | Switch.`dc` |
    | `static NetworkDatacenter` | NetDatacenterBroker.`linkDC` |

    Methods in org.cloudbus.cloudsim.network.datacenter with parameters of type NetworkDatacenter

    | Modifier and Type | Method and Description |
    | --- | --- |
    |  |  |
    | --- | --- |
    | `void` | NetDatacenterBroker.`setLinkDC(NetworkDatacenter alinkDC)` |

    Constructors in org.cloudbus.cloudsim.network.datacenter with parameters of type NetworkDatacenter

    Constructor and Description

    `AggregateSwitch(java.lang.String name,
    int level,
    NetworkDatacenter dc)`

    Constructor for Aggregate Switch We have to specify switches that are connected to its
    downlink and uplink ports, and corresponding bandwidths

    `EdgeSwitch(java.lang.String name,
    int level,
    NetworkDatacenter dc)`

    Constructor for Edge Switch We have to specify switches that are connected to its downlink
    and uplink ports, and corresponding bandwidths.

    `RootSwitch(java.lang.String name,
    int level,
    NetworkDatacenter dc)`

    Constructor for Root Switch We have to specify switches that are connected to its downlink
    ports, and corresponding bandwidths

    `Switch(java.lang.String name,
    int level,
    NetworkDatacenter dc)`

- Overview
- Package
- Class
- Use
- Tree
- Deprecated
- Index
- Help

- Prev
- Next

- Frames
- No Frames

- All Classes
